# Glycerolized Li^+^ Ion Conducting Chitosan-Based Polymer Electrolyte for Energy Storage EDLC Device Applications with Relatively High Energy Density

**DOI:** 10.3390/polym12061433

**Published:** 2020-06-26

**Authors:** Ahmed S. F. M. Asnawi, Shujahadeen B. Aziz, Muaffaq M. Nofal, Muhamad H. Hamsan, Mohamad A. Brza, Yuhanees M. Yusof, Rebar T. Abdilwahid, Saifful K. Muzakir, Mohd F. Z. Kadir

**Affiliations:** 1 Chemical Engineering Section, Malaysian Institute of Chemical & Bioengineering Technology (UniKL MICET), Universiti Kuala Lumpur, Alor Gajah 78000, Malacca, Malaysia; asyafiq.asnawi@s.unikl.edu.my; 2 Hameed Majid Advanced Polymeric Materials Research Laboratory, Department of Physics, College of Science, University of Sulaimani, Qlyasan Street, Kurdistan Regional Government, Sulaimani 46001, Iraq; mohamad.brza@gmail.com (M.A.B.); rebar.abdulwahid@univsul.edu.iq (R.T.A.); 3 Department of Civil Engineering, College of Engineering, Komar University of Science and Technology, Kurdistan Regional Government, Sulaimani 46001, Iraq; 4 Department of Mathematics and General Sciences, Prince Sultan University, P.O. Box 66833, Riyadh 11586, Saudi Arabia; muaffaqnofal@gmail.com; 5 Institute for Advanced Studies, University of Malaya, Kuala Lumpur 50603, Malaysia; hafizhamsan93@gmail.com; 6 Department of Manufacturing and Materials Engineering, Faculty of Engineering, International Islamic University of Malaysia, Kuala Lumpur, Gombak 53100, Malaysia; 7 Malaysian Institute of Chemical and Bio-Engineering Technology, Universiti Kuala Lumpur (UniKL MICET), Alor Gajah 78000, Malacca, Malaysia; yuhanees@unikl.edu.my; 8 Material Technology Program, Faculty of Industrial Sciences & Technology, Universiti Malaysia Pahang, Lebuhraya Tun Razak, Gambang, Kuantan 26300, Pahang, Malaysia; saifful@ump.edu.my; 9 Centre for Foundation Studies in Science, University of Malaya, Kuala Lumpur 50603, Malaysia; mfzkadir@um.edu.my

**Keywords:** chitosan, glycerol plasticizer, electrical properties, impedance study, EDLC fabrication

## Abstract

In this study, the solution casting method was employed to prepare plasticized polymer electrolytes of chitosan (CS):LiCO_2_CH_3_:Glycerol with electrochemical stability (1.8 V). The electrolyte studied in this current work could be established as new materials in the fabrication of EDLC with high specific capacitance and energy density. The system with high dielectric constant was also associated with high DC conductivity (5.19 × 10^−4^ S/cm). The increase of the amorphous phase upon the addition of glycerol was observed from XRD results. The main charge carrier in the polymer electrolyte was ion as *t_el_* (0.044) < *t_ion_* (0.956). Cyclic voltammetry presented an almost rectangular plot with the absence of a Faradaic peak. Specific capacitance was found to be dependent on the scan rate used. The efficiency of the EDLC was observed to remain constant at 98.8% to 99.5% up to 700 cycles, portraying an excellent cyclability. High values of specific capacitance, energy density, and power density were achieved, such as 132.8 F/g, 18.4 Wh/kg, and 2591 W/kg, respectively. The low equivalent series resistance (*ESR*) indicated that the EDLC possessed good electrolyte/electrode contact. It was discovered that the power density of the EDLC was affected by *ESR*.

## 1. Introduction

Renewable energy and high-performance energy devices are required for the human lifestyle because of the increasing demand for a clean environment [1]. Recently, an electrochemical double-layer capacitor (EDLC) emerges as a potential alternative for conventional batteries and even fuel cells. The principle of energy storage mechanism in this device is based on the non-Faradaic process, where the ions accumulate at the interfacial region in the form of a double-layer [2]. This means only charge accumulation occurs between the electrode surfaces and the bulk electrolyte, and there is no electron transfer (i.e., Faradaic process). Based on the relatively high power density, durability, and thermal stability, EDLC is superior over supercapacitors. It is also taken into consideration that the cost-effectiveness and straightforwardness of the fabrication of EDLC are of significant importance in terms of technological aspects [2,3,4]. For EDLC fabrication, the electrodes have been made from several materials, such as graphite [5], carbon aerogel [6], carbon nanotubes [7], and activated carbon [8]. The activated carbon is intensively and extensively utilized as characterized by a large surface area, satisfactory chemical stability, and high electronic conductivity [9]. Another component in EDLC design is the electrolyte between the electrodes where solid polymer electrolytes (SPEs) with conductivity between ~10^−4^ and 10^−3^ Scm^−1^ have been used. A few attempts have been introduced to improve the conductivity of such electrolytes, for instance, salt impregnation and plasticization [10,11]. Gupta et al. [12] studied the conductivity of hydroxyl ethyl cellulose film, which was increased up to 3.8 × 10^−5^ Scm^−1^ and 4.4 × 10^−3^ Scm^−1^ with the addition of 0.5 wt.% lithium tetraborate (Li_2_B_4_O_7_) salt and 20 wt.% glycerol, respectively. In another study, there has been an increase in conductivity of dextran-ammonium nitrate (NH_4_NO_3_) system from 3.00 ± 1.60 × 10^−5^ Scm^−1^ to 1.15 ± 0.08 × 10^−3^ Scm^−1^ with 20 wt.% of glycerol addition [13]. The critical step in increasing conductivity depends upon the plasticization process, which facilitates the degree of dissociation of salts [14]. The liquid electrolytes are widely used because of their high performance in various energy devices despite the ease of evaporation and leakage, which causes corrosion [15,16]. However, the SPEs have several advantages compared to liquid electrolyte counterparts, such as safety and ease of fabrication, as well as long shelf life [17]. There are two categories of polymer hosts—natural and synthetic polymers. These are commonly documented in the synthesis of SPEs that are used in electrochemical energy devices [18]. The synthetic polymers are non-biodegradable that impact petroleum resources; hence, it is detrimental to the environment [19]. In terms of biodegradability, the synthetic polymers as a polymer host in the study of energy storage devices have been utilized over the last decades. Thereby, less plastic waste pollutants will release into the environment. The natural polymers and biopolymers are used interchangeably, are typically characterized by cost-effectiveness, high compatibility with solvents, high capability in film-forming, and natural abundance [20,21]. For example, starch, cellulose, and carrageenan are most commonly used as polymer hosts [22,23,24]. Another common biopolymer that is extensively under intensive investigation in energy storage devices is chitosan [25]. This biopolymer enriches in various oxygen-containing functional groups (i.e., availability of non-bonding electron pair) based on chemical structure. The problem of low conductivity in chitosan is solved by salt incorporation, where the mechanism of conduction is significantly affected. The ions from the incorporated salts are in sufficient contact with functional groups within the polymer body that provide dative bond [26]. Recently, it has been shown that the addition of glycerol accompanying specific lithium salt into chitosan-based polymer electrolyte improves conductivity. Such a conducting electrolyte system has been employed in EDLC fabrication.

## 2. Materials and Methods

### 2.1. Materials

In the present work, chitosan with a relatively high molecular mass of around 310,000 to 375,000 g/mol along with glycerol in the fabrication of plasticized systems was used as received. Both chemicals were purchased from Sigma-Aldrich (Missouri, MO, USA).

### 2.2. Polymer Electrolyte Preparation

The procedure involved the addition of 1 g of Chitosan (CS) into 50 mL of acetic acid (1%) solution. Afterward, a constant weight ratio (40 wt.%) of lithium acetate (LiCO_2_CH_3_) salt was added to the previous solution. The solution was stirred continuously with the magnetic stirrer at room temperature until a homogenous solution was obtained. Then, a various weight percentage ratio of glycerol was added to the polymer-salt mixture, with stirring continuously until a clear solution was gained. The percentage of glycerol content in the prepared samples varied from 14 to 42 wt.%. The samples were then coded as CSGL1, CSGL2, and CSGL3 for CS:LiCO_2_CH_3_ electrolyte incorporated with 14 wt.%, 28 wt.%, and 42 wt.% of glycerol, respectively. Finally, the solutions were then spilled into different clean and dry glass Petri dishes, covered with filter paper to prevent any contamination. The Petri dishes were left to evaporate solvent slowly at room temperature, to obtain dry and a free-standing plasticized CS polymer electrolyte films.

### 2.3. Impedance and X-Ray Diffraction (XRD) Studies

To investigate the electrical properties of the films, the electrochemical impedance spectroscopy (EIS) [HIOKI, 3532–50 LCR HiTESTER, Hioki, Nagano, Japan] from 50 Hz to 5 MHz was employed for receiving the impedance spectra of the samples. D5000 X-ray diffractometer (1.5406 Å)[ Bruker AXS GmbH, Berlin, Germany] was used for the structural study of the polymer films. The scanning angle of the films was set in the range of 10° to 80° (resolution = 0.1°).

### 2.4. Transference Number (TNM) and Linear Sweep Voltammetry (LSV) Measurements

**Figure 1:**
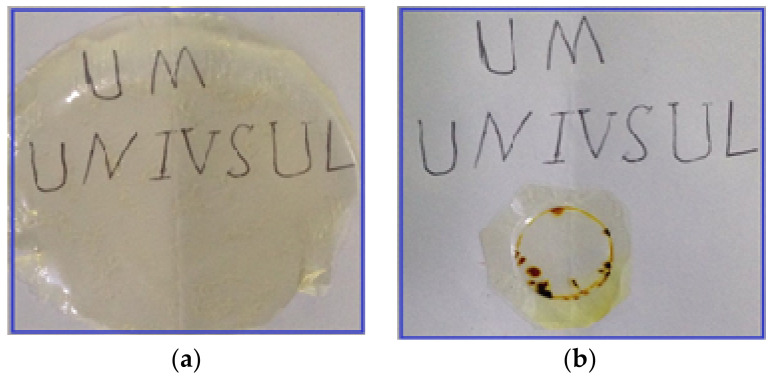
(**a**) Image of the fabricated solid polymer electrolyte (SPE). (**b**) Circle image of the fabricated SPE after linear sweep voltammetry (LSV) examination.

The digital DC power supply, V&A Instrument (Neware, Shanghai, China) DP3003, was employed to perform the transference number (TNM) analysis from the DC polarization technique. Before starting measurements, the relatively high conducting electrolyte was sandwiched between blocking stainless steel electrodes in a Teflon holder. The electrode polarization was carried out by holding potential at 0.8 V, and the DC current was recorded as a function of time at room temperature. The potential window of the conducting electrolyte was also determined using linear sweep voltammetry (LSV) analysis (DY2300 potentiostat, Neware, Shenzhen, China) at a scan rate of 10 mV/s. The image of the fabricated SPE is indicated in Figure 1a, and the circle image of the SPE after LSV examination is shown in Figure 1b. It is seen in Figure 1b that the SPE film was oxidized beyond the decomposition voltage.

### 2.5. EDLC Preparation

The electrode made of polyvinylidene fluoride (PVdF), activated carbon, and carbon black materials was used in the EDLC study. The detail of the electrode preparation can be seen in our previous work [2]. The thickness of the electrodes varied to obtain an optimum of 25 μm. The specific capacitance (*C_s_*) was calculated from the CV response using the following relation [27]:

(1) $$C_{spe}=\int_{V_{i}}^{V_{f}}\frac{I\left(V\right)dV}{2m\upsilon \left(V_{2}-V_{1}\right)}$$

where ʃ *I(V)dV* is the area of the CV curve, which was determined using Origin 9.0 software; *m* and *v* are the mass of active material and scan rate, respectively. The potential range of 0.9 V to 0 V from starting (*V*_2_) to the ending (*V*_1_), respectively, was applied. The charge-discharge profiles of the system were tested using a Neware battery cycler with a current density of 0.5 mA/cm^2^. The specific capacitance (*C_s_*) from charge-discharge profiles and equivalent series resistance (*ESR*) were calculated using the Equations shown below [28,29]:

(2) $$C_{s}=\frac{i}{sm}$$

(3) $$ESR=\frac{V_{d}}{i_{}}$$

where *i* is the applied current, *s* is the slope of discharge part, and *V_d_* is the voltage drop.

## 3. Results and Discussion

### 3.1. Dielectric Properties

**Figure 2:**
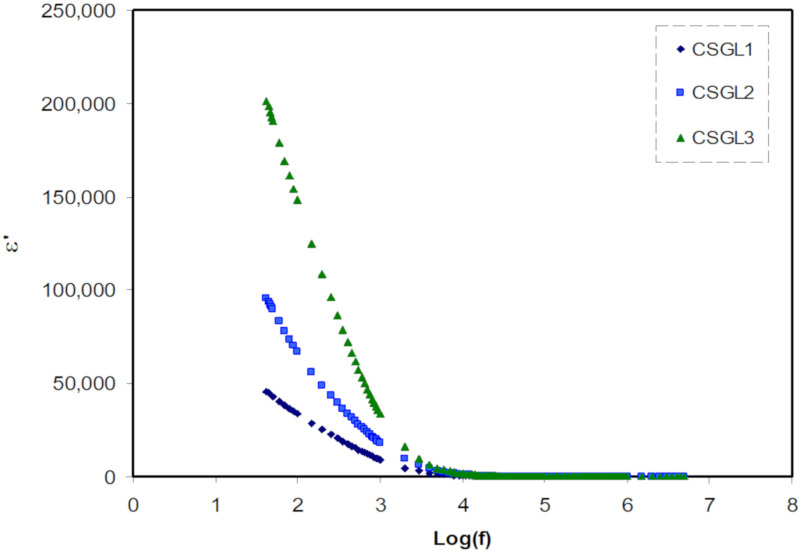
Dielectric constant versus log (f) for all polymer blend electrolytes.

**Figure 3:**
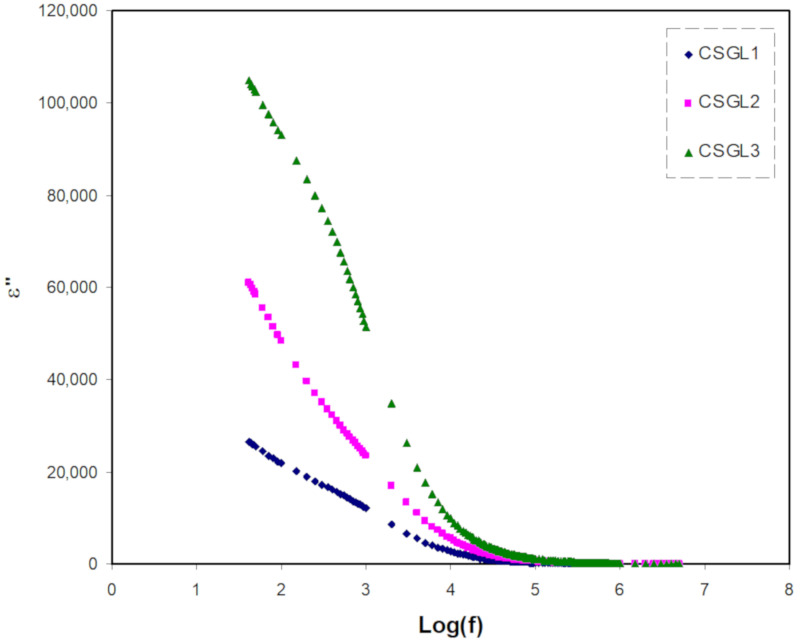
Dielectric loss versus log (f) for all polymer blend electrolytes.

To deal with ionic transport phenomenon in the SPEs, it is of the best choice to analyze the dielectric relaxation. From this analysis, one can examine the nature and ionic movement of polymer-plasticizer interactions [30,31]. To tackle dielectric properties, there are several parameters, such as relative permittivity, loss tangent, dielectric constant, microwave reflection coefficient, split post dielectric resonance technique, and terahertz material [31]. The dielectric constant or complex permittivity is defined by the following mathematical relation [32]:

(4) (ε*)=ε′−jε″

where ε′ is the real dielectric constant, and ε″ is the imaginary dielectric loss, which essentially specifies the energy loss and storage in every cycle of the applied power supply [30]. Both the real and imaginary parts of complex permittivity (ε^*^) are calculated from the impedance data (i.e., *Z′* and *Z″*) using the Equations presented below [33,34],

(5) $$\varepsilon \prime =\left[\frac{Z\prime \prime }{\omega C_{o}({Z\prime }^{2}+{Z\prime \prime }^{2})}\right]$$

(6) $$\varepsilon \prime \prime =\left[\frac{Z\prime }{\omega C_{o}({Z\prime }^{2}+{Z\prime \prime }^{2})}\right]$$

where *ω* is the angular frequency of the applied filed (*ω* *= 2πf*), and ε′ and ε″ are the dielectric constant and dielectric loss, respectively. The *C*_o_ is the vacuum capacitance given by *ε*_o_*A*/*t*, *ε*_o_ is the permittivity of free space, *A* is the electrode cross-sectional area, and *t* is the film thickness. Figure 2 and Figure 3 show the real and imaginary part of dielectric constant (ε′, ε″) as a function of frequency at room temperature. It is observable that the value of ε′ rises very sharply for the high amount of glycerol plasticizer at the low-frequency region as a result of the influence of space charge and electrode polarization (EP) [35]. At the electrode/electrolyte interfacial region, a high concentration of charge carriers accumulated because of the increase in the polarization in the low frequency [36]. At the higher frequency regions, it is clearly seen that there is no possibility of excess ion dispersion to align with the direction of the field as the consequence of the periodic reversal of the electric field takes place rapidly. Regarding both dielectric constant and dielectric loss, there is a decrease in the values due to the polarity declination [37]. It is known that the carrier density is in strong association with the dissociation energy (U) as well as dielectric constant (ɛ′), which can be formulated in this relationship (*n* = *n*_o_ exp(−*U*/ɛ′*KT*)).

The value of dielectric constant increases with the increasing salt concentration and there is a substantial increase in the number of charge carriers and thus a rise in DC conductivity [38,39,40,41]. To investigate and comprehend the SPEs’ conductivity behavior, it is best to measure DC conductivity and dielectric constant, as well as a function of the concentration of the salt at room temperature.

### 3.2. Impedance Study

Impedance spectroscopy is a powerful technique to be used in examining the ionic conductivity of polymeric materials [42,43,44]. Electrochemical impedance spectroscopy (EIS) is also used as an impressive technique to analyze electrical properties of the new materials that are utilized in electrochemical energy devices, for example, double-layer capacitance, diffusion layer, and charge transfer resistance [39].

**Figure 4:**
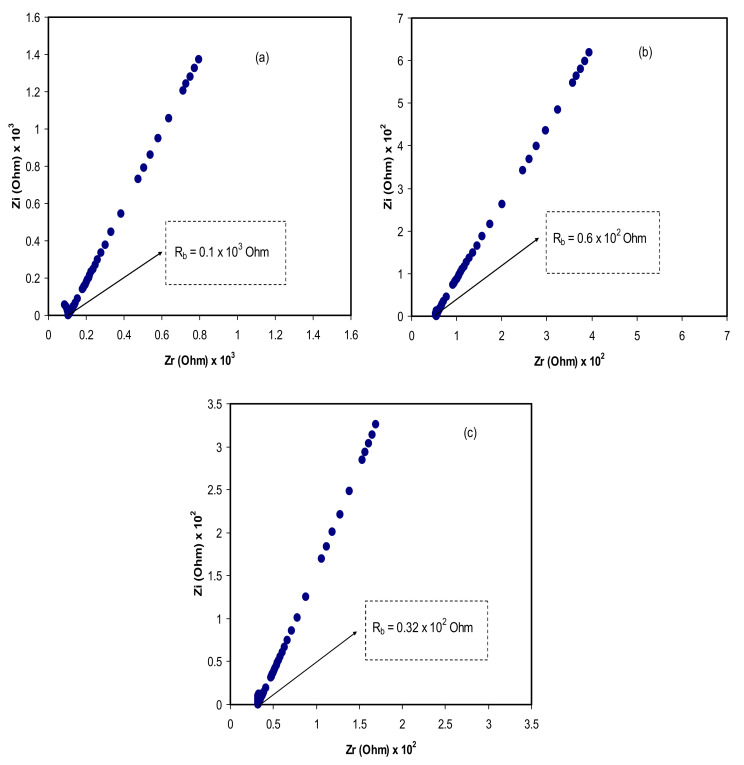
Complex impedance plots for (**a**) CSGL 1, (**b**) CSGL 2, and (**c**) CSGL 3.

Over the previous decade, the ion-conducting membrane is a class of materials, which have attracted attention owing to the wide applications in solid electrochemical devices [42]. The EIS responses of polymer electrolytes commonly consist of a semicircle and a spike at high and low-frequency regions, respectively [43]. The semicircle results from charge transfer at the interface. On the contrary, similar behavior is not obtained in the measurement of the EIS, as presented in Figure 4a–c. At the low-frequency region, the spike region arises owing to EP (that is, the effect of blocking electrodes). The EP phenomenon occurs as a result of the growth of electric double-layer, as a consequence of free charge accumulation at the solid electrolyte and electrode interface [45,46]. Accordingly, at the low frequency, it is supposedly for the complex impedance to show a straight line parallel to the imaginary axis. In other words, the straight line’s inclination ought to be 90°, and the blocking double-layer capacitance (EP phenomena) at the blocking electrodes is responsible for the inclination [47,48]. The ionic conductivity of the CS:LiCO_2_CH_3_:Gly systems can be calculated using the following Equation [49]:

(7) $$\sigma_{dc}=\left(\frac{1}{R_{b}}\right)\times \left(\frac{t}{A}\right)$$

where *t* is the thickness of the sample, *R_b_* is the bulk resistance of the material, and *A* is the area of the electrode [50,51]. It is of vital importance to determine the DC conductivity of the samples from the *R_b_* value using the above Equation.

The DC conductivities are calculated and tabulated, as shown in Table 1. It is seen that the highest ionic conductivity is recorded for lower bulk resistance *R_b_* material, with 5.19 × 10^−4^ Scm^−1^. Therefore, one reason behind employing polymer blend electrolytes in practical usage in electrical double-layer capacitors (EDLCs) is a relatively high DC conductivity. The dependency of conductivity of electrolytes on the number density and the mobility of the ions is well-known and has been mathematically shown [40,50]:

(8) σ=∑ηqμ

where *ƞ* denotes the carrier density, *q* denotes simple charge, and *μ* represents mobility. An earlier study has suggested that the charge species of Li^+^ ion is released by lithium salt within the polymer-lithium salt system [51]. Table 1 shows the DC conductivity values of electrolyte samples at ambient temperature. From Figure 4, it is seen that there is a decrease in the bulk resistance with increasing plasticizer concentration from 14 to 42 wt.%. It has also been confirmed that it is essential for polymer electrolytes to be used in electrochemical device applications, such as batteries and EDLCs, if the DC conductivity lies between 10^−5^ and 10^−3^ S cm^−1^ [52,53]. It is worth-mentioning that glycerol is helpful in improving DC conductivity in polymer electrolytes when plasticized. The more insight into the influence of glycerol as a plasticizer in polymer electrolytes has been discussed in the next section.

### 3.3. XRD Analysis

It is known that in the preparation of a polymer electrolyte, pure polymer materials consist of a mixture of amorphous and crystalline phases [41,51]. It is of great importance to determine the degree of crystallinity in a polymer structure using an accurate method. Therefore, the determination of crystallinity is not only useful in determining the molecular structure of a crystalline polymer but also in comprehensive understanding and rationalizing the intrinsic properties of polymeric materials [31]. In the previous work, it has been emphasized that chitosan (CS) possesses a range of crystalline peaks centered at 2θ° = 15° and 20° in the XRD pattern. The rigid crystalline structure of chitosan is mainly kept via hydrogen bonding, including intermolecular and intramolecular [51].

**Figure 5:**
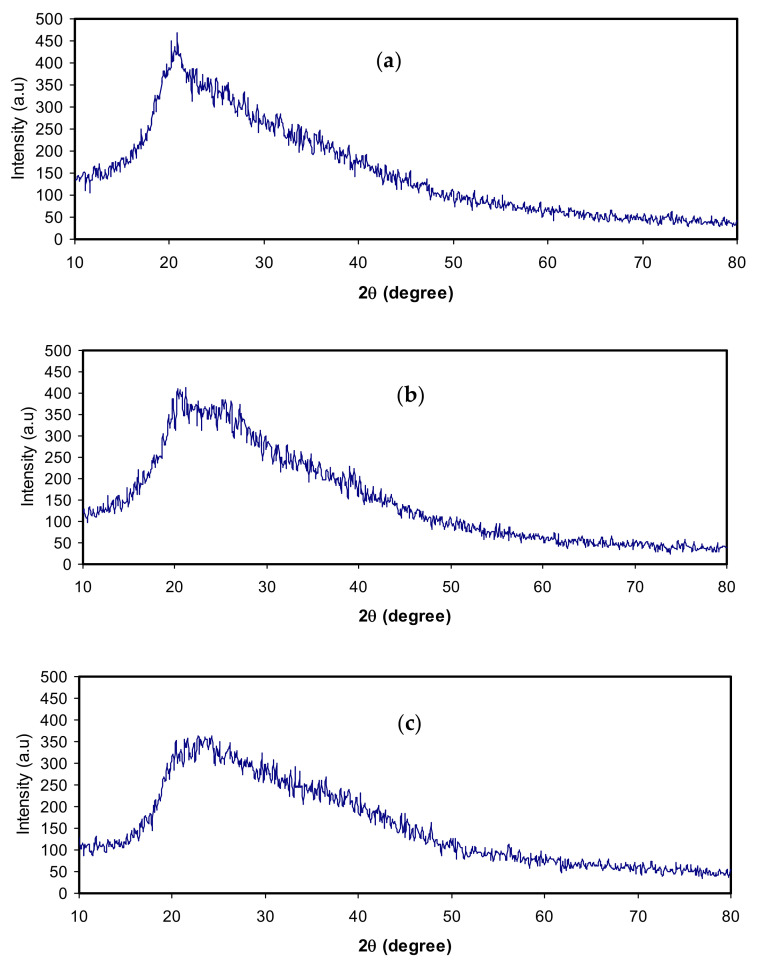
XRD pattern for (**a**) CSGL 1, (**b**) CSGL 2, and (**c**) CSGL 3.

The XRD pattern for CS:LiCO_2_CH_3_ plasticized with various quantities of glycerol as a plasticizer is shown in Figure 5. It is clearly seen that with increasing glycerol, the peak intensity decreases, and the broadness increases. Earlier studies have confirmed that plasticizer incorporation to polymer electrolytes is helpful for increasing both the conductivity and the amorphous phase [5,8,11]. Plasticizers also reduce the number of active centers, thereby weakening the intermolecular and intramolecular forces between the polymer chains. Consequently, the reduction in the degree of crystallinity makes the salt dissociation capability to be guaranteed, and as a consequence, an enhancement of charge carrier transport occurs [11]. Based on these observations, the increase in DC conductivity observed in impedance analysis (see Table 1) may be related to the increase in amorphous as well as the dissociation of salts.

### 3.4. TNM Study

**Figure 6:**
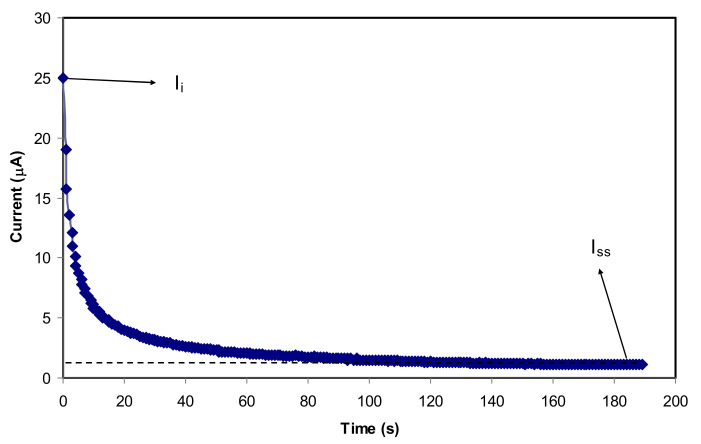
Current versus time for the highest conducting electrolyte.

The transference number measurement (TNM) is performed to evaluate the conductivity of the glycerolized system. Figure 6 exhibits the plot of current against time during polarization for the relatively high conducting electrolyte (CSGL 3). Shukur et al. [54] stated that the ionic conductivity within the polymer electrolyte system is responded by ions if the transference number of ion (*t_ion_*) is close to unity. The transference number of the ion and electron (*t_el_*) is calculated using the following Equations [55].

(9) $$t_{ion}=\frac{I_{i}-I_{ss}}{I_{i}}$$

(10) $$t_{el}=1-t_{ion}$$

where *I_i_* and *I_ss_* represent the current at initial and steady-state, respectively.

Figure 6 shows current time transient, where the initial current decreases over electrochemical course due to the decrease in ionic species in the electrolyte and reaches a steady state when it is completely reduced [56]. Under steady-state conditions, the movement of mobile ions is balanced by the diffusion process [56]. Rani et al. [57] stated that during the polarization process, the stainless steel electrodes are responsible for the current flow blocking of ions as a result of the passing of electrons only through the solid metallic electrodes.

Using Equations (9) and (10), the values of *t_ion_* and *t_el_* for the electrolyte under study are 0.956 and 0.044, respectively. This result indicates the dominancy of ions for conducting in this electrolyte. Mohan et al. [58] reported that a high *t_ion_* value of 0.98 was obtained for a relatively high conducting electrolyte in the poly (vinyl chloride) (PVC)—poly (ethyl methacrylate) (PEMA)—sodium perchlorate (NaClO_4_) system. Shukur et al. [59] reported that the plasticized system of chitosan-ammonium bromide (NH_4_Br) gave the *t_ion_* and *t_el_* values of 0.98 and 0.02, respectively. Thus, the present results for the electrolyte system are comparable to those reported in the literature. However, in the previous reports [60,61], the low values of the transference number of Li^+^ ions have been recorded for the unplasticized systems of Lithium bis (fluorosulfonyl) imide/poly (ethylene oxide) (PEO) polymer and polyacrylonitrile (PAN)/PEO/organo-modification of the SWy-2 (org-SWy) electrolytes. In our work, glycerol provides more ions for the electrolyte systems, causing an increment in the ionic transference number.

### 3.5. Linear Sweep Voltammetry (LSV)

**Figure 7:**
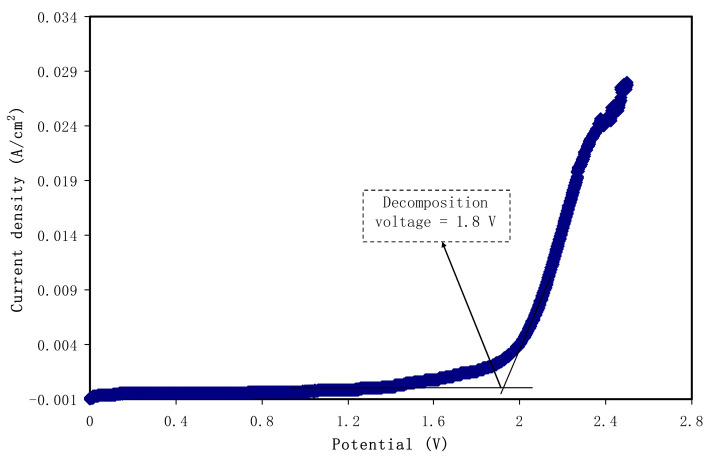
Linear sweep voltammetry (LSV) curve for the highest conducting electrolyte.

The potential window of the electrolyte at room temperature can be determined from the LSV study. The LSV response from 0 to 2.5 V for the relatively high conducting electrolyte (CSGL 3) at 10 mV/s is presented in Figure 7. It is observed that there is no noticeable current flow at the potential below 1.80 V, indicating the absence of electrochemical reactions [62]. The final potential (i.e., decomposition) of the window of the electrolyte ends at 1.80 V, which is comparable with the value documented for the plasticized PEMA-based polymer electrolyte system by Anuar et al. [63]. Therefore, the decomposition potential obtained for the electrolyte under study is highly satisfactory to be applied in EDLC that normally operates at 1.0 V [64].

### 3.6. CV and EDLC Characterization

**Figure 8:**
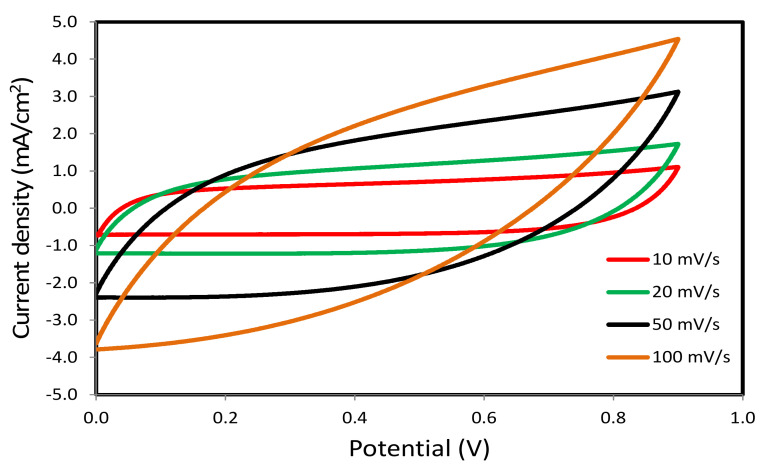
Cyclic voltammetry (CV) curves of the fabricated electrochemical double-layer capacitor (EDLC) at different scan rates.

The cyclic voltammetry (CV) is carried out on the carbon electrode and chitosan (CS):LiCO_2_CH_3_:Glycerol to evaluate the capacitive behavior of the EDLC under study. The CV is carried out at different scan rates, as shown in Figure 8. It is interesting to note that the CV shape turns from leaf-shape to rectangular as the scan rate is decreased. To explain this phenomenon, the factor of the type of electrodes is crucial because the difference in internal resistance and porosity causes the shape of CV response to be an imperfect rectangular shape [65]. Of special interest, no peak is observed, suggesting no possibility of a Faradaic process (i.e., charge transfer) in the fabricated EDLC. Instead, both cations and anions in the EDLC migrate to negative and positive electrodes, respectively, during the charging process. The anion is attracted by the positive electrode, while the opposite situation occurs at the negative electrode. The high electric field holds the ions and electrons by electrolyte and electrode, respectively [66]. This indicates the development of a double-layer charge at the surface of carbon electrodes, where the energy is stored in the form of potential energy [67]. The values of *C_s_* at each scan rate are calculated from the CVs using Equation (1) and are tabulated in Table 2.

**Figure 9:**
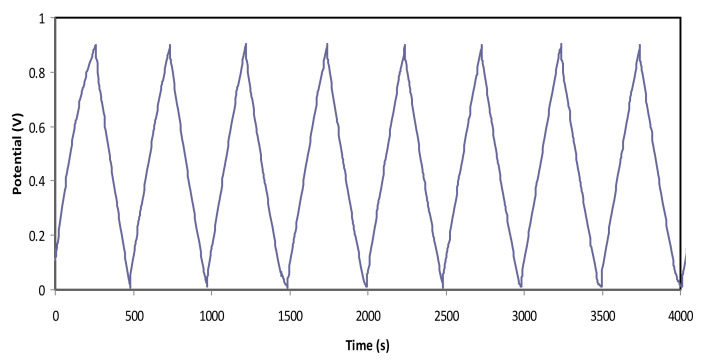
Charge-discharge profile for the fabricated EDLC device.

As the scan rate is increased, the *C_s_* value is seen to decrease. It results in lowering the number of stored charges on the surface of the electrodes at a high scan rate, which, in turn, results in the increment of energy loss and decrease in the *C_s_* values [68]. To examine the charge-discharge process in the EDLC under the study, the galvanostatic technique is applied. Figure 9 exhibits the charge-discharge profile of the EDLC on the carbon electrode and chitosan (CS):LiCO_2_CH_3_:Glycerol. The linear capacitive behavior in the EDLC is verified via the discharge slope [52]. The *C_s_* of the EDLC from charge-discharge curves is calculated using Equation (2).

**Figure 10:**
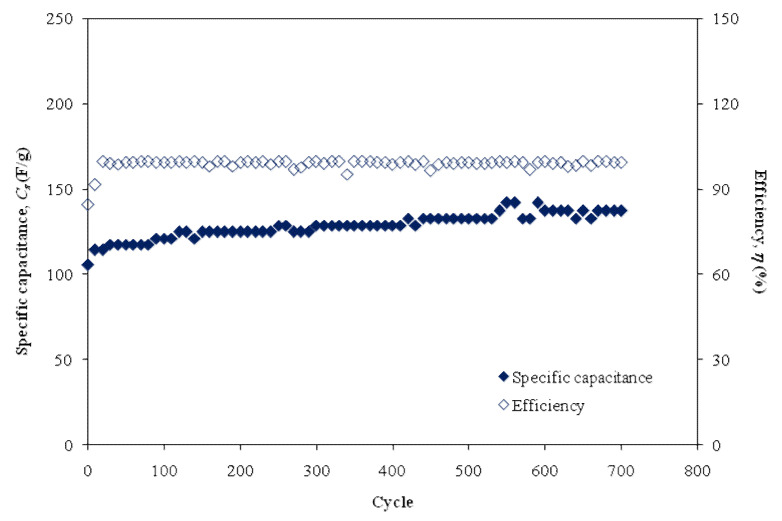
Specific capacitance, *C_s_,* and efficiency, *η*, of the fabricated EDLC for 700 cycles.

Figure 10 shows the calculated *C_s_* values of the EDLC over a long electrochemical course of 700 cycles. At the 1st cycle, the *C_s_* is found to be 105.5 F/g, which is slightly different compared to that extracted from CV analysis, which proves that EDLC exhibits the characteristics of the capacitor cell [69]. At the 400th cycle, the *C_s_* increases to 132.8 F/g and becomes almost constant at an average value of 136.8 F/g up to the 700th cycle. An incredibly interesting observation is that the *C_s_* obtained in this study is comparable to the *C_s_* value for the fabricated EDLC, which was at ~130 F/g, as documented by Yadav et al. [70]. Consequently, it is suitable to use plasticized chitosan (CS): LiCO_2_CH_3_:Glycerol electrolyte as a new material in the fabrication of EDLC with high specific capacitance. It is also critical to know the columbic efficiency (*η*) parameter regarding the cycling stability of the EDLC, where it is calculated from the following Equation [13].

(11) $$\eta_{}=\frac{t_{d}}{t_{c}}\times 100$$

where *t_d_* and *t_c_* are the discharge and charge time, respectively. Figure 10 shows the efficiency of the fabricated EDLC, where plasticized chitosan (CS):LiCO_2_CH_3_:Glycerol is used over 700 cycles. At the 1st cycle, the efficiency is found to be 84.6% and increases to 91.4% at the 10th cycle. Importantly, at the 30th cycle, the efficiency is observed to be 98.8% and then kept constant at ~99.5% up to 700 cycles. This indicates plausible electrode-electrolyte contact as the efficiency is higher than 90.0% EDLC under study [71].

**Figure 11:**
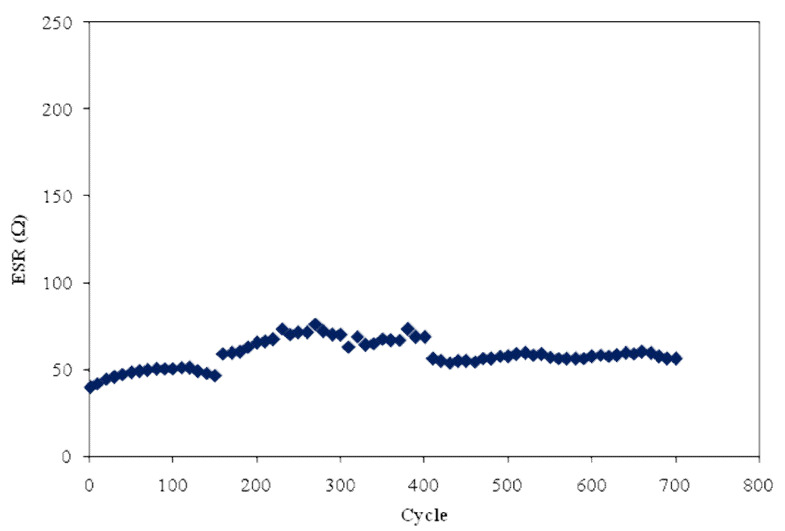
Equivalent series resistance (ESR) of the fabricated EDLC for 700 cycles.

From the charge-discharge profile in Figure 9, there are tiny potential drops (*V_d_*) before the discharging process begins. This can be related to the existence of internal resistance in the EDLC, which is called equivalent series resistance (*ESR*). This *ESR* of the EDLC can be obtained from Equation (3). Figure 11 exhibits the *ESR* of the EDLC for 700 cycles. It can be determined that the *ESR* value varies from 40 to 75 Ω over 700 cycles, and it is noticeable that the value is slightly increased with a positive slope throughout the 700 cycles. As mentioned by Arof et al. [72], the internal resistance exists due to the charge and discharge process of electrolytes, type of current collectors (aluminum foils), and also the gap between electrolyte and electrode. The small value of *ESR* portrays good contact between the electrode and the electrolyte and indicates that it is easy for ions to migrate toward the surface of the electrode to form an electrical double-layer [73]. A similar trend is observed for the starch-lithium acetate system, where the *ESR* is slightly increased when the *C_s_* remains at a constant value [74]. Besides, the rapid charging and discharging process will lead to the recombination of free ions, and then the ion pair will be developed, which leads to the conductivity decrement. Kang et al. [75] also stated that *ESR* is strongly related to the conductivity of an electrolyte.

The energy density (*E*) and power density (*P*) are also important to describe the performance of the EDLC. These parameters can be expressed by using the following Equations [76].

(12) $$E=\frac{C_{s}V^{2}}{2}$$

(13) $$P_{}=\frac{V^{2}}{4m(ESR)}$$

**Figure 12:**
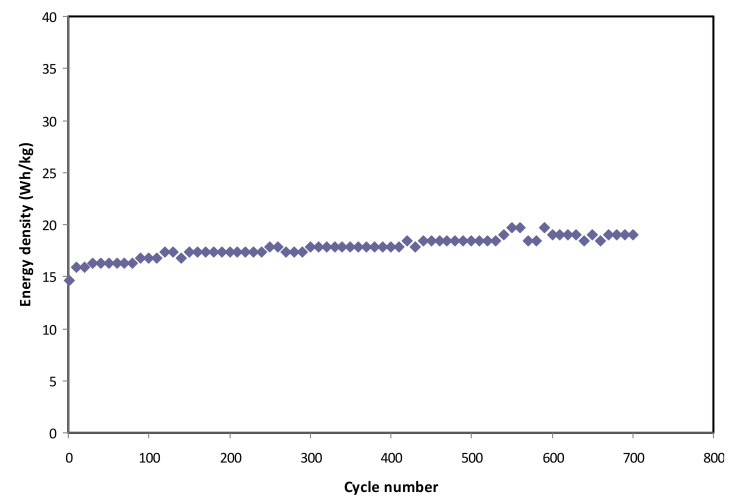
Energy density, *E*, of the fabricated EDLC for 700 cycles.

**Figure 13:**
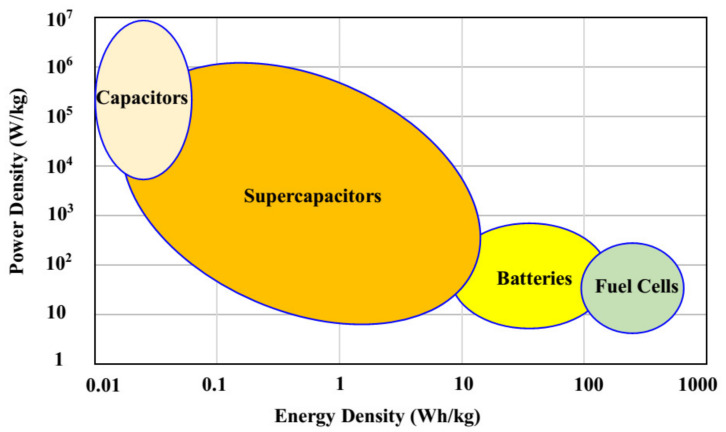
Ragone plot of energy density versus power density for numerous electrical energy storage and conversion devices [83].

**Figure 14:**
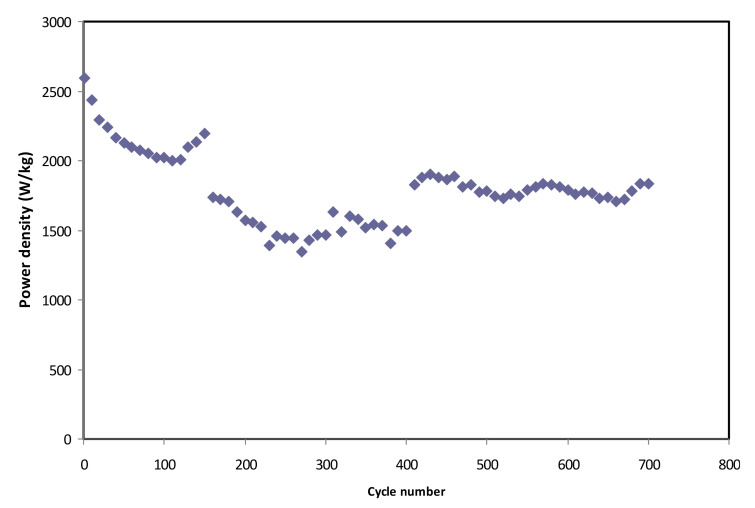
The power density of the fabricated EDLC for 100 cycles.

Figure 12 exhibits the calculated energy density for 700 cycles. The EDLC obtains the energy density (*E*) of 14.7 Wh/kg at the 1st cycle, and the value is gradually increased to 18.4 Wh/kg at the 450th cycle and then kept stable at 19.1 Wh/kg from 540th cycle towards the final cycle. This trend is harmonized with the pattern of *C_s_* illustrated in Figure 10. This result explains that the charge carriers require almost the same amount of energy to migrate towards the surface of the electrodes for the entire process of charge and discharge [77]. The results of the present work reveal that biopolymer-based electrolytes are crucial for energy storage applications. Previous studies have indicated that biopolymer-based electrolytes have been widely utilized in electrochemical devices, such as EDLC and batteries [78,79,80,81,82], but their electrochemical performances are still low. In the present work, high energy density (19.1 Wh/kg) is obtained, which can be considered as a new approach in this field. EDLCs are devices, which can occupy the spaces between electrochemical batteries and electrostatic capacitors with regard to energy density and power density [83]. The achieved energy density in the current work is 19.1 Wh/kg, which is in the range of the energy density of batteries (see Figure 13). Thus, the challenges in this field of study (EDLC study) are to design EDLC devices with an energy density close enough to batteries in addition to its high power density. Moreover, the power density (*P*) values are calculated by using Equation (13) for selected cycles, where *P* value at the 1st cycle is found to be 2.591 × 10^3^ W/kg (see Figure 14). Then, it is dropped to 1830.6 W/kg at the 400th cycle and has remained constant until the EDLC has completed 700 cycles. The trend of *P* is in agreement with the trend of the *ESR* plot. This is because the depletion of electrolytes occurs when the internal resistance increases, causing the recombination of ions due to the fast charging and discharging mechanism, thus resulting in reduced *P* at a high cycle number [84]. Both *E* and *P* values are clearly dependent on the mass loading of active material in the fabrication of EDLC. The low mass loading and relatively low current are reported to be responsible for providing enhanced electrochemical performance [85].

## 4. Conclusions

Chitosan (CS):LiCO_2_CH_3_:Glycerol polymer electrolytes were prepared with a solution cast method. The electrolyte studied in this current work could be established as new materials in the fabrication of EDLC with high specific capacitance, energy density, and power density. The dielectric properties were correlated with DC conductivity to understand the conductivity behavior of the plasticized electrolytes. The system with high dielectric constant was also associated with high DC conductivity (5.19 × 10^−4^ Scm^−1^). The reduction of crystallinity and increase of DC conductivity upon the addition of glycerol were ascribed to the disruption of intermolecular hydrogen bonds and dissociation of more salts through the electrolyte. Li^+^ and CH_3_CO_2_^-^ ions were the main charge carriers throughout the conduction process rather than electrons as *t_el_* was less than *t_ion_*. These relatively high values of both ion transference number and potential stability confirmed the possibility of the fabricated systems for the electrochemical device as energy storage. The sharp drop in the current value of the TNM plot verified that the CS:LiCO_2_CH_3_:Glycerol was an ionic conductor. From CV analysis, it was found that the specific capacitance reduced from 77.36 F/g to 21.89 F/g as the scan rate changed from 10 mV/s to 100 mV/s, respectively. The capacitive characteristic of the fabricated EDLC was confirmed as no redox peaks were observed in the CV plot, as well as the linearity of the discharge curve. The EDLC was stable up to 700 cycles, where the efficiency was stable from 98.8% to 99.5%. Ions in CS:LiCO_2_CH_3_:Glycerol was considered to have almost the same energy barrier during the conduction process as the energy density (average *E* = 18.4 Wh/kg) was almost constant throughout 700 cycles. The specific capacitance of the EDLC was 132.8 F/g. The power density of the EDLC was quite high (2591 W/kg). This could be due to the low value of ESR, which was 40 Ω.

## Figures and Tables

**Table 1:** Calculated DC conductivity for CS:LiCO_2_CH_3_:Gly electrolyte films at room temperature.

Sample Designation	DC Conductivity (S/cm)
CSGL1	1.66 × 10^−4^
CSGL2	2.77 × 10^−4^
CSGL3	5.19 × 10^−4^

**Table 2:** Specific capacitance (*C_s_*) of the electrochemical double-layer capacitor (EDLC) at different scan rates.

Scan Rate (mV/s)	Specific Capacitance, *C_s_* (F/g)
10	77.36
20	60.73
50	37.85
100	21.89
